# Supplementary material for: Modeling glioblastoma heterogeneity as a dynamic network of cell states
Source: Mol Syst Biol. 2021 Sep 16;17(9):e10105. doi: 10.15252/msb.202010105 (PMC8444284; doi:10.15252/msb.202010105)
Supplement: Supplementary file 5 — Source Data for Figure 3 [file MSB-17-e10105-s001.zip › Figure3A_sourcedata/GSEA_3065/hallmarks_state5.GseaPreranked.1623416302988/HALLMARK_MYC_TARGETS_V1.html]

Details for gene set HALLMARK\_MYC\_TARGETS\_V1[GSEA]

|  || Dataset | state5 |
| Phenotype | NoPhenotypeAvailable |
| Upregulated in class | na\_neg |
| GeneSet | HALLMARK\_MYC\_TARGETS\_V1 |
| Enrichment Score (ES) | -0.7103971 |
| Normalized Enrichment Score (NES) | -2.6149309 |
| Nominal p-value | 0.0 |
| FDR q-value | 0.0 |
| FWER p-Value | 0.0 |
Table: GSEA Results Summary

  

Fig 1: Enrichment plot: HALLMARK\_MYC\_TARGETS\_V1      
 Profile of the Running ES Score & Positions of GeneSet Members on the Rank Ordered List

  

| PROBE | GENE SYMBOL | GENE\_TITLE | RANK IN GENE LIST | RANK METRIC SCORE | RUNNING ES | CORE ENRICHMENT || 1 | CANX |  |  | 53 | 0.634 | 0.0051 | No |
| 2 | PRDX4 |  |  | 80 | 0.553 | 0.0119 | No |
| 3 | XPO1 |  |  | 933 | 0.220 | -0.0812 | No |
| 4 | HNRNPA2B1 |  |  | 1852 | 0.122 | -0.1834 | No |
| 5 | IFRD1 |  |  | 2073 | 0.107 | -0.2066 | No |
| 6 | HNRNPU |  |  | 2204 | 0.098 | -0.2196 | No |
| 7 | TUFM |  |  | 2244 | 0.095 | -0.2224 | No |
| 8 | TRA2B |  |  | 2285 | 0.093 | -0.2253 | No |
| 9 | DDX18 |  |  | 2288 | 0.093 | -0.2239 | No |
| 10 | SRSF1 |  |  | 2334 | 0.090 | -0.2275 | No |
| 11 | NOP56 |  |  | 2390 | 0.086 | -0.2322 | No |
| 12 | SRSF2 |  |  | 2908 | 0.057 | -0.2900 | No |
| 13 | SRSF7 |  |  | 3126 | 0.046 | -0.3139 | No |
| 14 | RFC4 |  |  | 3212 | 0.041 | -0.3228 | No |
| 15 | CAD |  |  | 3232 | 0.039 | -0.3243 | No |
| 16 | CTPS1 |  |  | 3314 | 0.036 | -0.3329 | No |
| 17 | NCBP2 |  |  | 3487 | 0.028 | -0.3520 | No |
| 18 | PSMC6 |  |  | 3744 | 0.018 | -0.3808 | No |
| 19 | ORC2 |  |  | 3888 | 0.012 | -0.3968 | No |
| 20 | SRPK1 |  |  | 4294 | -0.006 | -0.4428 | No |
| 21 | DHX15 |  |  | 4397 | -0.010 | -0.4542 | No |
| 22 | CDK2 |  |  | 4489 | -0.013 | -0.4643 | No |
| 23 | MCM4 |  |  | 4534 | -0.015 | -0.4691 | No |
| 24 | CDC45 |  |  | 4563 | -0.017 | -0.4720 | No |
| 25 | XPOT |  |  | 4624 | -0.019 | -0.4785 | No |
| 26 | RNPS1 |  |  | 4641 | -0.020 | -0.4799 | No |
| 27 | MYC |  |  | 4656 | -0.020 | -0.4812 | No |
| 28 | DDX21 |  |  | 4735 | -0.023 | -0.4896 | No |
| 29 | MCM2 |  |  | 4824 | -0.027 | -0.4992 | No |
| 30 | NCBP1 |  |  | 4901 | -0.029 | -0.5073 | No |
| 31 | PABPC4 |  |  | 4947 | -0.031 | -0.5119 | No |
| 32 | TARDBP |  |  | 4963 | -0.032 | -0.5130 | No |
| 33 | MCM7 |  |  | 4986 | -0.032 | -0.5150 | No |
| 34 | TOMM70 |  |  | 5004 | -0.033 | -0.5163 | No |
| 35 | GOT2 |  |  | 5108 | -0.037 | -0.5274 | No |
| 36 | SF3B3 |  |  | 5151 | -0.038 | -0.5315 | No |
| 37 | SNRPA |  |  | 5242 | -0.042 | -0.5410 | No |
| 38 | CSTF2 |  |  | 5250 | -0.042 | -0.5410 | No |
| 39 | SF3A1 |  |  | 5538 | -0.054 | -0.5727 | No |
| 40 | BUB3 |  |  | 5596 | -0.057 | -0.5782 | No |
| 41 | CCNA2 |  |  | 5671 | -0.061 | -0.5855 | No |
| 42 | KPNA2 |  |  | 5811 | -0.066 | -0.6002 | No |
| 43 | ABCE1 |  |  | 6018 | -0.075 | -0.6223 | No |
| 44 | PWP1 |  |  | 6126 | -0.081 | -0.6330 | No |
| 45 | G3BP1 |  |  | 6152 | -0.082 | -0.6344 | No |
| 46 | CUL1 |  |  | 6216 | -0.086 | -0.6401 | No |
| 47 | PSMD14 |  |  | 6275 | -0.089 | -0.6451 | No |
| 48 | UBE2E1 |  |  | 6278 | -0.089 | -0.6438 | No |
| 49 | RRM1 |  |  | 6298 | -0.090 | -0.6444 | No |
| 50 | EIF4A1 |  |  | 6340 | -0.092 | -0.6474 | No |
| 51 | RUVBL2 |  |  | 6371 | -0.093 | -0.6492 | No |
| 52 | PSMD1 |  |  | 6431 | -0.097 | -0.6542 | No |
| 53 | AIMP2 |  |  | 6494 | -0.101 | -0.6594 | No |
| 54 | SMARCC1 |  |  | 6547 | -0.105 | -0.6635 | No |
| 55 | MCM5 |  |  | 6553 | -0.105 | -0.6622 | No |
| 56 | EIF3B |  |  | 6559 | -0.105 | -0.6610 | No |
| 57 | MAD2L1 |  |  | 6600 | -0.108 | -0.6636 | No |
| 58 | HSP90AB1 |  |  | 6611 | -0.109 | -0.6628 | No |
| 59 | NOP16 |  |  | 6691 | -0.113 | -0.6698 | No |
| 60 | CBX3 |  |  | 6756 | -0.118 | -0.6750 | No |
| 61 | PRPS2 |  |  | 6830 | -0.123 | -0.6812 | No |
| 62 | APEX1 |  |  | 6898 | -0.129 | -0.6865 | No |
| 63 | COPS5 |  |  | 6916 | -0.130 | -0.6862 | No |
| 64 | CDC20 |  |  | 6985 | -0.136 | -0.6915 | No |
| 65 | CCT2 |  |  | 7013 | -0.138 | -0.6922 | No |
| 66 | POLE3 |  |  | 7101 | -0.146 | -0.6995 | No |
| 67 | KPNB1 |  |  | 7144 | -0.150 | -0.7016 | No |
| 68 | MRPS18B |  |  | 7173 | -0.153 | -0.7021 | No |
| 69 | TFDP1 |  |  | 7187 | -0.154 | -0.7009 | No |
| 70 | RAD23B |  |  | 7194 | -0.155 | -0.6989 | No |
| 71 | EIF4E |  |  | 7292 | -0.166 | -0.7070 | No |
| 72 | EIF3J |  |  | 7306 | -0.167 | -0.7055 | No |
| 73 | SYNCRIP |  |  | 7350 | -0.175 | -0.7073 | Yes |
| 74 | EIF2S1 |  |  | 7366 | -0.177 | -0.7059 | Yes |
| 75 | MCM6 |  |  | 7375 | -0.178 | -0.7037 | Yes |
| 76 | SNRPA1 |  |  | 7376 | -0.178 | -0.7006 | Yes |
| 77 | USP1 |  |  | 7398 | -0.181 | -0.6998 | Yes |
| 78 | PSMD3 |  |  | 7409 | -0.183 | -0.6977 | Yes |
| 79 | MRPL9 |  |  | 7446 | -0.186 | -0.6985 | Yes |
| 80 | CCT3 |  |  | 7464 | -0.187 | -0.6972 | Yes |
| 81 | HNRNPA3 |  |  | 7470 | -0.188 | -0.6944 | Yes |
| 82 | MRPL23 |  |  | 7516 | -0.193 | -0.6962 | Yes |
| 83 | RRP9 |  |  | 7517 | -0.193 | -0.6928 | Yes |
| 84 | FAM120A |  |  | 7521 | -0.193 | -0.6897 | Yes |
| 85 | GNL3 |  |  | 7548 | -0.197 | -0.6892 | Yes |
| 86 | EXOSC7 |  |  | 7567 | -0.200 | -0.6878 | Yes |
| 87 | STARD7 |  |  | 7579 | -0.201 | -0.6855 | Yes |
| 88 | NOLC1 |  |  | 7699 | -0.218 | -0.6952 | Yes |
| 89 | PSMA2 |  |  | 7701 | -0.219 | -0.6914 | Yes |
| 90 | SNRPB2 |  |  | 7704 | -0.219 | -0.6878 | Yes |
| 91 | AP3S1 |  |  | 7728 | -0.223 | -0.6865 | Yes |
| 92 | ETF1 |  |  | 7748 | -0.225 | -0.6847 | Yes |
| 93 | PHB |  |  | 7753 | -0.226 | -0.6812 | Yes |
| 94 | FBL |  |  | 7780 | -0.230 | -0.6801 | Yes |
| 95 | CLNS1A |  |  | 7824 | -0.239 | -0.6808 | Yes |
| 96 | EIF3D |  |  | 7833 | -0.241 | -0.6775 | Yes |
| 97 | PRPF31 |  |  | 7837 | -0.242 | -0.6736 | Yes |
| 98 | HDAC2 |  |  | 7865 | -0.247 | -0.6723 | Yes |
| 99 | PSMA4 |  |  | 7883 | -0.251 | -0.6698 | Yes |
| 100 | SSB |  |  | 7906 | -0.257 | -0.6678 | Yes |
| 101 | IMPDH2 |  |  | 7948 | -0.266 | -0.6678 | Yes |
| 102 | HPRT1 |  |  | 7974 | -0.271 | -0.6659 | Yes |
| 103 | ACP1 |  |  | 7982 | -0.272 | -0.6619 | Yes |
| 104 | PRDX3 |  |  | 8005 | -0.277 | -0.6595 | Yes |
| 105 | UBA2 |  |  | 8067 | -0.290 | -0.6614 | Yes |
| 106 | PCNA |  |  | 8082 | -0.293 | -0.6578 | Yes |
| 107 | SSBP1 |  |  | 8096 | -0.297 | -0.6541 | Yes |
| 108 | PSMA1 |  |  | 8109 | -0.299 | -0.6502 | Yes |
| 109 | DUT |  |  | 8118 | -0.300 | -0.6458 | Yes |
| 110 | VDAC1 |  |  | 8120 | -0.300 | -0.6406 | Yes |
| 111 | CCT5 |  |  | 8125 | -0.302 | -0.6358 | Yes |
| 112 | HDDC2 |  |  | 8129 | -0.303 | -0.6308 | Yes |
| 113 | TCP1 |  |  | 8137 | -0.306 | -0.6262 | Yes |
| 114 | VDAC3 |  |  | 8151 | -0.309 | -0.6222 | Yes |
| 115 | CYC1 |  |  | 8152 | -0.309 | -0.6168 | Yes |
| 116 | PSMC4 |  |  | 8153 | -0.309 | -0.6114 | Yes |
| 117 | TRIM28 |  |  | 8170 | -0.318 | -0.6076 | Yes |
| 118 | SRSF3 |  |  | 8191 | -0.323 | -0.6042 | Yes |
| 119 | PHB2 |  |  | 8199 | -0.326 | -0.5992 | Yes |
| 120 | CCT4 |  |  | 8211 | -0.329 | -0.5947 | Yes |
| 121 | ILF2 |  |  | 8220 | -0.331 | -0.5898 | Yes |
| 122 | HNRNPR |  |  | 8231 | -0.334 | -0.5851 | Yes |
| 123 | EIF4H |  |  | 8247 | -0.338 | -0.5808 | Yes |
| 124 | LDHA |  |  | 8263 | -0.343 | -0.5765 | Yes |
| 125 | PCBP1 |  |  | 8291 | -0.351 | -0.5734 | Yes |
| 126 | PSMB2 |  |  | 8292 | -0.352 | -0.5672 | Yes |
| 127 | GSPT1 |  |  | 8299 | -0.355 | -0.5617 | Yes |
| 128 | EIF2S2 |  |  | 8314 | -0.358 | -0.5570 | Yes |
| 129 | PA2G4 |  |  | 8320 | -0.360 | -0.5512 | Yes |
| 130 | XRCC6 |  |  | 8325 | -0.361 | -0.5453 | Yes |
| 131 | HDGF |  |  | 8330 | -0.362 | -0.5394 | Yes |
| 132 | EIF1AX |  |  | 8331 | -0.363 | -0.5330 | Yes |
| 133 | NDUFAB1 |  |  | 8333 | -0.363 | -0.5268 | Yes |
| 134 | GLO1 |  |  | 8338 | -0.364 | -0.5208 | Yes |
| 135 | PSMD7 |  |  | 8343 | -0.368 | -0.5148 | Yes |
| 136 | PPM1G |  |  | 8376 | -0.380 | -0.5117 | Yes |
| 137 | RSL1D1 |  |  | 8388 | -0.385 | -0.5062 | Yes |
| 138 | VBP1 |  |  | 8395 | -0.387 | -0.5001 | Yes |
| 139 | NAP1L1 |  |  | 8404 | -0.389 | -0.4942 | Yes |
| 140 | HNRNPC |  |  | 8413 | -0.392 | -0.4882 | Yes |
| 141 | CCT7 |  |  | 8416 | -0.392 | -0.4815 | Yes |
| 142 | HSPD1 |  |  | 8418 | -0.393 | -0.4747 | Yes |
| 143 | SNRPD3 |  |  | 8423 | -0.395 | -0.4682 | Yes |
| 144 | HNRNPD |  |  | 8436 | -0.402 | -0.4625 | Yes |
| 145 | LSM2 |  |  | 8445 | -0.405 | -0.4563 | Yes |
| 146 | PGK1 |  |  | 8452 | -0.408 | -0.4498 | Yes |
| 147 | RPS10 |  |  | 8475 | -0.421 | -0.4449 | Yes |
| 148 | UBE2L3 |  |  | 8506 | -0.438 | -0.4406 | Yes |
| 149 | POLD2 |  |  | 8508 | -0.439 | -0.4330 | Yes |
| 150 | SNRPD1 |  |  | 8537 | -0.451 | -0.4283 | Yes |
| 151 | RAN |  |  | 8555 | -0.463 | -0.4221 | Yes |
| 152 | EEF1B2 |  |  | 8557 | -0.463 | -0.4140 | Yes |
| 153 | CNBP |  |  | 8565 | -0.466 | -0.4066 | Yes |
| 154 | HNRNPA1 |  |  | 8570 | -0.469 | -0.3989 | Yes |
| 155 | NME1 |  |  | 8573 | -0.471 | -0.3908 | Yes |
| 156 | PSMB3 |  |  | 8576 | -0.472 | -0.3827 | Yes |
| 157 | SNRPG |  |  | 8578 | -0.473 | -0.3745 | Yes |
| 158 | ERH |  |  | 8580 | -0.475 | -0.3663 | Yes |
| 159 | PTGES3 |  |  | 8587 | -0.479 | -0.3585 | Yes |
| 160 | PABPC1 |  |  | 8598 | -0.488 | -0.3511 | Yes |
| 161 | TYMS |  |  | 8599 | -0.488 | -0.3425 | Yes |
| 162 | C1QBP |  |  | 8600 | -0.490 | -0.3339 | Yes |
| 163 | PSMD8 |  |  | 8619 | -0.507 | -0.3270 | Yes |
| 164 | CDK4 |  |  | 8625 | -0.510 | -0.3186 | Yes |
| 165 | EIF4G2 |  |  | 8627 | -0.510 | -0.3098 | Yes |
| 166 | YWHAE |  |  | 8629 | -0.512 | -0.3009 | Yes |
| 167 | SLC25A3 |  |  | 8637 | -0.517 | -0.2926 | Yes |
| 168 | SERBP1 |  |  | 8642 | -0.520 | -0.2839 | Yes |
| 169 | NHP2 |  |  | 8649 | -0.524 | -0.2753 | Yes |
| 170 | HSPE1 |  |  | 8650 | -0.524 | -0.2661 | Yes |
| 171 | NPM1 |  |  | 8706 | -0.566 | -0.2624 | Yes |
| 172 | SNRPD2 |  |  | 8718 | -0.581 | -0.2535 | Yes |
| 173 | TXNL4A |  |  | 8728 | -0.591 | -0.2441 | Yes |
| 174 | YWHAQ |  |  | 8730 | -0.592 | -0.2338 | Yes |
| 175 | LSM7 |  |  | 8762 | -0.621 | -0.2264 | Yes |
| 176 | DEK |  |  | 8770 | -0.627 | -0.2162 | Yes |
| 177 | COX5A |  |  | 8785 | -0.639 | -0.2065 | Yes |
| 178 | RPL14 |  |  | 8787 | -0.642 | -0.1953 | Yes |
| 179 | SET |  |  | 8813 | -0.664 | -0.1865 | Yes |
| 180 | RACK1 |  |  | 8814 | -0.664 | -0.1748 | Yes |
| 181 | RANBP1 |  |  | 8819 | -0.669 | -0.1635 | Yes |
| 182 | PSMA7 |  |  | 8821 | -0.670 | -0.1518 | Yes |
| 183 | ODC1 |  |  | 8839 | -0.697 | -0.1415 | Yes |
| 184 | RPL22 |  |  | 8874 | -0.739 | -0.1324 | Yes |
| 185 | RPL34 |  |  | 8877 | -0.744 | -0.1195 | Yes |
| 186 | SRM |  |  | 8880 | -0.746 | -0.1066 | Yes |
| 187 | RPLP0 |  |  | 8904 | -0.777 | -0.0956 | Yes |
| 188 | RPL18 |  |  | 8907 | -0.780 | -0.0821 | Yes |
| 189 | PPIA |  |  | 8913 | -0.797 | -0.0687 | Yes |
| 190 | RPS6 |  |  | 8929 | -0.837 | -0.0556 | Yes |
| 191 | RPS3 |  |  | 8936 | -0.850 | -0.0414 | Yes |
| 192 | RPL6 |  |  | 8942 | -0.856 | -0.0269 | Yes |
| 193 | RPS5 |  |  | 8946 | -0.865 | -0.0120 | Yes |
| 194 | RPS2 |  |  | 8960 | -0.923 | 0.0027 | Yes |
Table: GSEA details [plain text format]

  

Fig 2: HALLMARK\_MYC\_TARGETS\_V1: Random ES distribution      
 Gene set null distribution of ES for **HALLMARK\_MYC\_TARGETS\_V1**
